# Barriers and facilitators to providing home-based care in a pandemic: policy and practice implications

**DOI:** 10.1186/s12877-022-02907-w

**Published:** 2022-03-21

**Authors:** Sue Anne Bell, Lydia Krienke, Allyson Brown, Jen Inloes, Zoe Rettell, Tamar Wyte-Lake

**Affiliations:** 1 School of Nursing, University of Michigan, Ann Arbor, MI USA; 2 Johns Hopkins University School of Nursing, Baltimore, MD USA; 3 University of Michigan, Ann Arbor, MI USA; 4 Veterans Emergency Management Evaluation Center, North Hills, CA USA

**Keywords:** Home-based care, Disaster, COVID-19, Policy, Practice

## Abstract

**Objective:**

The purpose of this study is to describe the experiences of home-based care providers (HBCP)  in providing care to older adults during the pandemic in order to inform future disaster planning, including during pandemics.

**Design:**

Qualitative inquiry using an abductive analytic approach.

**Setting and participants:**

Home-based care providers in COVID-19 hotspots.

**Methods:**

Telephone interviews were conducted with 27 participants (administrators, registered nurses and other members of the allied healthcare team), who provided in-home care during the pandemic in Medicare-certified home health agencies. Interviews focused on eliciting experiences from HBCP on challenges and successes in providing home-based care to older adults, including barriers to care and strategies employed to keep patients, and providers, safe in their homes during the pandemic.

**Results:**

Data was distilled into four major themes that have potential policy and practice impact. These included disrupted aging-in-place resources, preparedness actions contributing to readiness for the pandemic, limited adaptability in administrative needs during the pandemic and challenges with unclear messaging from public health officials.

**Conclusions:**

Home-based care plays an essential role in maintaining the health of older adults in disaster contexts, including pandemics. Innovative solutions, informed by policy that generate evidence-based best practices to support HBCP are needed to reduce barriers and increase protective factors, in order to maintain continuity of care for this vulnerable population during disruptive events.

## Introduction

The COVID-19 pandemic generated new, and exacerbated existing, concerns for home-based care providers (HBCP) and their clients. In the midst of confusion around best infection control measures and safety against this novel virus, home-based care leadership and staff turned to the Centers for Disease Control and Prevention (CDC) and local public health authorities for answers [1, 2]. Especially at the onset of the pandemic, home-based care organizations questioned the best actions to take in order to keep their patients and their staff healthy and safe, while organizations also faced financial losses from changes in billing and unexpected PPE costs [1–4]. In addition, the uncertainty around COVID-19 pandemic left patients and providers fearful for their own health [5, 6]. Though the CDC initially put forth guidance in May of 2020 – with updated guidelines in October 2020 – for how home-based care agencies could operate safely amidst a pandemic [7], additional strategies to help this workforce remain functional during novel disasters, such as pandemics, remain needed at both a local and policy level [8].

Home-based care is defined as the broad range of services provided in the home to support patients, which can include both caregiving and personal care services, as well as skilled services (e.g. nursing and therapy) [9]. This study draws from the experiences of the subset of home-based care defined as Medicare-certified home health agencies. These home health agencies provide skilled care in the home, most often through inter-disciplinary care teams, and play a critical role in linking their patients to their surrounding healthcare system [10]. And although some Home-based care providers, such as home health agency staff, only work with their patients short-term, they broadly serve a unique role in educating, monitoring, and supporting the holistic needs of patients, and it has been demonstrated that they can play a critical role during disasters such as hurricanes and fires [11, 12]. Given the importance of home-based care and its growing role, there remains a young body of literature around the experiences of these providers during disasters and pandemics. Especially needed is knowledge around the successful workarounds employed by HBCP, and the barriers to supporting clients, particularly those with policy and practice implications. Therefore, the purpose of this study was to describe the experiences of HBCP, specifically among Medicare-certified home health agencies in providing care to older adults during a novel pandemic in order to inform future disaster planning.

## Methods

This descriptive, qualitative study was part of a parent study that explored experiences of providing in-home care during a disaster, from a sample of providers working in Medicare-certified home health agencies. The study was expanded to include HBCP experiences during a pandemic, informing the larger study by providing evidence from a unique, national-level disaster. The study was informed by the social-ecological model [13]. Institutional Review Board (IRB) approval was received from the University of Michigan (HUM00132531). This study adhered to the consolidated criteria for reporting qualitative research (COREQ) guidelines.

### Interview guide

An interview guide informed by prior conceptual work on disasters, home care, and aging was developed [14–18]. This guide was initially refined through pilot testing with qualitative experts and registered nurses. Interviews focused on the experience of providing home-based care to older adults during the COVID-19 pandemic, including barriers to care and strategies employed to keep patients safe in their homes. Questions investigated factors contributing to exacerbations of chronic medical conditions, unplanned hospital admissions, and other breakdowns in quality patient care. The interview guide questions were modified minimally from the broader definition of a disaster to that of a pandemic. The final interview guide was constructed to facilitate a goal interview length of up to 60 min.

### Study sample and recruitment

Data from the Johns Hopkins Coronavirus Resource Center World Map was used in order to identify US counties with the greatest number of reported COVID-19 cases at the time of the study [19]. A list of Medicare-certified home health agencies that participate in Medicare programs was generated using an open-source database [20]. Agencies located in counties with higher numbers of confirmed positive COVID-19 cases were prioritized, meaning that they were called first by the research team. At the time, these were in New York, Illinois, and Michigan.

A two-stage sampling design was employed. First, we contacted via telephone the 370 agencies that met the study criteria. Each agency was contacted via telephone by a member of the study team up to five times. After a fifth unanswered call or request for a call back, the agency was removed from the study due to non-response. Once contact was made, the study was explained to the agency administrator or their representative, and study information was disseminated to potential participants. In order to obtain a broad representation of the various roles of the interdisciplinary HHA teams, recruitment within each program was extended to include both administrator-level staff as well as any member of the clinical team. Individuals then contacted the study team if they were interested in participating. Recruitment of a diverse sample of home-based care providers remains a methodological challenge. We focus on the strength of data adequacy in this study, using purposive sampling to reach a targeted sample size, as described in a systematic review of sample size sufficiency in relation to saturation in qualitative studies [21]. We consider the low response rate to our recruitment calls acceptable given that home-based care provider priorities were focused on patient care during a crisis rather than research participation. For this reason, we accepted the limitations of recruitment during an active pandemic and are grateful to the study participants for their time spent with us.

### Individual interviews

Individual interviews were conducted over by telephone from August through October of 2020. Participants resided within counties in New York, Illinois, and Michigan. Counties are not identified in this study in order to protect confidentiality. Each individual interview was conducted by a trained member of the study team who was responsible for audio recording, informed consent and incentive documents, and note-taking. Interviews lasted approximately 60 min and began with an introduction to encourage participants to speak openly, followed by obtaining verbal informed consent, after which the semi-structured interview was initiated.

### Analysis

The processes of coding and analysis were initiated after the study team reached consensus through weekly debriefing meetings that data had reached saturation, guided by expert literature on saturation [22, 23]. Individual interview conversations were recorded digitally and transcribed by an IRB-approved transcription service. After removing identifying information from the transcripts, including the names of specific facilities, other clinicians, or family members, the transcripts were formatted for coding. Data was analyzed using an abductive analytic approach [24]. This method combines inductive and deductive analysis thereby allowing for a purposeful examination of a range of explanations, which reduces the likelihood of bias. Using an iterative process, four research assistants independently generated codes using an organizing framework that applied the four stages of the disaster life cycle’s (mitigation, preparedness, response, and recovery) to the social-ecological model [25]. Coders met over the video conference platform Zoom for Health weekly in order to review and arbitrate differences in each other’s codes. The final codes and their agreed upon definitions were then entered into a codebook. A total of 30 codes were generated from the data. After systematic analysis and collective deliberation, four themes emerged from the data that represented larger overarching concepts. These themes represent common strategies used by participants as well as the barriers they faced in providing care to older adults during the COVID-19 pandemic.

## Results

### Demographics

A total of 27 participants were individually interviewed, representing eight counties in the states of New York, Illinois, and Michigan. Table 1 summarizes participant characteristics.

**Table 1:** Participant characteristics

Characteristic	n (%)
Male Gender	8 (30)
Age	
18–30	2 (7)
31–45	7 (26)
46–60	13 (48)
61–75	5 (18)
Race	
Black	4 (15)
White	10 (37)
Hispanic/Latinx	1 (4)
Asian	12 (44)
Role on care team^a^	
Caregiver/Home health aide	5 (18)
Physical Therapist	1 (4)
Registered nurse	10 (37)
Licensed practical nurse	1 (4)
Administrator	14 (52)
Length of time in occupation (years)	
0–5	6 (22)
6–10	5 (18)
11–20	7 (26)
21 +	9 (33)
Highest level of education	
High school	1 (4)
Associate’s degree	4 (15)
Bachelor’s degree	15 (55)
Master’s degree	5 (18)
Doctorate	2 (7)

^a^May report more than one role

### Themes

Four themes were generated from the analysis specific to policy and practice. The themes represent facilitators and barriers to providing home-based care for older adults before and during the COVID-19 pandemic.

#### Theme 1: disrupted aging-in-place resources resulted in HBCP trying to close the gap

HBCP described observing patients’ health deteriorating due to the absence of social supports that they relied on prior to the pandemic. The pandemic-induced loss of these support networks placed patients’ mental and physical health at risk. This disruption in social networks was noted particularly around regular caregiving and community supports, especially formal or informal caregiving that supplemented the home-based care they received. Patients without strong social support networks were observed to be at greater risk for health breakdown during the COVID-19 pandemic.

“[A]t least one patient has been admitted to the hospital due to that she lives alone in a communal living building for seniors. Because of the pandemic they had completely taken out all social interaction, so there was no congregating between the other people who live in the building. There was no social eating. There was none of the games that they normally play. She's already isolated. She has not much family. There's nobody there to help her with a lot of things like groceries that she also depending on neighborly help for. During that time, she was in her home by herself and after several months of being there by herself… She deteriorated to the point where she had multiple falls, and nobody knew about them due to her not seeing any family or having anybody and refusing nursing staff.” P20 M1

“The unfortunate thing is when a caregiver that is not their family member and goes to several patients' homes ends up getting sick, then they're completely cut off from everything. Their health deteriorates because they're not able to take the medication that the caregiver was there in the home to help them with. They're isolated from getting the proper nutrition. They're not being fed fresh fruits or any of the nutrition that they normally would take or getting the medications that they need to take.” P20 M3

HBCP described the numerous activities of daily living, including day-to-day support with meals, repositioning, and household chores conducted on a daily basis by formal and informal caregivers, pre-pandemic. And why, with the loss of these supports, the HBCP team had to intensify their own efforts to support these gaps. Respondents described having to spend more time with patients making sure patients found a way to receive healthy meals, ensure medications were accessible and taken correctly, find creative ways to encourage patients to be physically active, and providing pandemic education.

“That’s my job, I think, as a home health provider. I had to do my best to take care of the patient because they didn’t want to go to the hospital. They didn’t want to go to anywhere. They just want to stay home. They tell us, “Okay. Try take care of me at home,” and I try to find ways to get it done. That’s what it is.” P22, N3

“Anything that we could, educating the patients over the phone and the families and reassuring them, letting them know, even sending them the guidance straight off of the CDC website about what was recommended for the PPE for the nurses to wear in the home and reassuring the families and also keeping it to the same staff member going in each time to see the patient instead of different faces. Continuity of care was paramount to gaining the patient’s trust.” P28 M5

The lack of community infrastructure to fall back on was exacerbated due to the pandemic.

“I think if a situation like that there should be a lot of more other organizations, not only home health, who would check on elderly people, in general, because a lot of patients—people, older—they don’t really have much service, or they don’t have any relatives. They need to be checked, bring food sometimes, even that. Pandemics, it needs to be volunteer organizations for the elderly, just to check on them if they need anything.” P11 G7

#### Theme 2: preparedness contributed to readiness for the pandemic

A number of participants viewed their prior training as a contributing factor to their preparedness during the pandemic. They described how frequent contact with patients with infectious conditions prior to the COVID-19 pandemic contributed to a sense of mastery in infection control practices. The education they received prior to the onset of the pandemic provided them with foundational knowledge in infection control practices, particularly around use of personal protective equipment (PPE) and protocols intended to minimize the spread of infection such as hand hygiene.

“I felt like home health was the biggest health sector that was the most prepared because we are trained. We are followed by Medicare once a year [...] They literally follow us into a patient’s home from start to finish. They meet us at the car. They follow us, and they see how we do things. A lot of it is that the clean technique, and the handwashing, and everything.... That part, I felt like I was more prepared than Dr. Fauci. I felt like, ‘Home health rocks. They rocked it.’ I think that’s why there wasn’t a lot of disease spread in the people’s homes because that part, we’ve had, we’ve been training on, and we’ve been tested on constantly.” P9 G2

“In home health, [we have] always used infection control. We were all prepared, and we were always prepared in teaching patients about infection control in their own homes and how to dispose of their dressings and things like that, right? In the community and in the patients' home we were good at that, and we were prepared for a pandemic.” P18 G3

Some agencies even reported having a stockpile in place due to previous experience with communicable disease outbreaks.

“PPEs, if you remember back maybe three, four, or five years ago, during the Ebola outbreak, we were prepared. We were able to stack up on gowns, face shields, masks, N95 masks, and that sort. Fortunately, we didn’t have to use those at that time, and when this COVID came in, we were more or less prepared. We were able to actually anticipate it a little bit, placed orders for PPEs back in late January, February, up till March when those things were still available.” P17 G3

Yet, not all agencies, or providers felt as prepared. While some agencies did have a pandemic plan, providers were either not familiar with its details or felt their infection control plan fell short given the unknown needs of this novel pandemic.

“Well, we didn’t have the proper PPE. The emergency preparedness plan didn’t even have a pandemic in it because when you create your emergency preparedness plan, you create it for things that could happen such as a snowstorm, a flood, a fire, a power outage. No one had, really, a really solid pandemic plan for their emergency preparedness plan. We never had a backup of N95s or regular even surgical masks to provide our nurses because it just was never something that we required or needed.” P28, N2

It was also noted that accessing pandemic resources, such as PPE, was a challenge. HBCP described not being well-connected or prioritized in terms of public health resources. Community-based organizations were found to be more available as a support to acquire PPE than government resources.

With being a home care provider, we weren’t high up on the supply chain list to get equipment, but without the equipment, we couldn’t walk into a patient’s house, so it was a real catch-22 to where I even had to reach out to a [religious] mission, who actually got us our first PPE equipment.” P5, M4

#### Theme 3: limited adaptability in administrative needs during the pandemic

Home-based care agencies and the patients they served experienced unprecedented financial concerns resulting from the pandemic, primarily around billing and insurance. Participants described limitations in receiving Medicare and Medicaid reimbursement and a lack of clarity in coverage guidelines. Additionally, they faced rapid changes in billing and reimbursement practices coupled with challenges in sourcing and purchasing PPE, leaving participants with confusion and uncertainty. HBCP described feeling abandoned and underappreciated by the federal government due to its failure to nimbly respond to the changing needs of HHAs during the pandemic.

“I don’t think Medicare realizes the role and what an important piece that home health agencies and hospices are all of the time, not just during a pandemic, but especially at times like these. I hope this draws attention to the importance of home health because Medicare has sure given us a hard time and cut back dollars and services, and making it very, very, very hard for home care agencies to survive financially with some of the cutbacks and things that they’ve done.” P21, E1

“The government came out with adjustments or flexibilities on the regulatory side, but we think that it wasn’t enough because we are still dealing with those up to this point. We have had patients who are not seen by the doctor in the office. [...] We’ve got to meet the face-to-face encounter requirement set up by Medicare. We are now being denied.[...] Up to this point, we are still having problems getting documentation to support services. A lot of cases in review got denied” P17 G2

Participants attributed HBCP agencies’ high-cost burden during the pandemic to a scarcity of PPE and lack of communication from governmental agencies surrounding reliable resources for obtaining PPE. This was particularly noted around the new and unexpected expense for PPE. Medicare reimbursement did not change to account for the fact that PPE was more expensive and being used in far greater quantities than before the pandemic.

“There are more expenses now being incurred for the care of patients under this present situation, whether it’s COVID or non-COVID. Before, the standard payment goes a long way. Now, the standard payment is almost the same with prior to the pandemic. We now entail more costs because of these PPEs so that we can also protect our staff as well as patients.” P10 G1

In some cases, the agencies themselves took on financial losses as a result:

“I was just hoping that the government would probably take into consideration the situation that we are in… Unfortunately, they are not going do anything about that. As a result, coverage is being denied, so we are not getting paid. We provided the services, but we are not going to get paid a single cent.” P17 N1

#### Theme 4: unclear messaging from public health officials

HBCP and their patients’ experienced frustration around the lack of clear and consistent information from public health officials on health, safety, and well-being during the novel pandemic. The delayed timing and execution of pandemic messaging hindered both providers’ and patients’ preparedness. Patients were confused about what information to believe because the news and social media changed rapidly and were often contradictory. HBCP tried their best to clarify information to patients, as demonstrated here:

“I think the preparation of the patients really coming from the news, social media, but since the inception of this pandemic, again, we started educating them also on the COVID, on social distancing, on infection control measures, on home environment sanitation. I think this was now reinforced to the patient, but again, most of them that they hear are all from social media. It’s a different thing when nurses and therapists start doing these instructions as part of their regular visits.” P10 G3

“Yeah, I think the most frustrating thing was the guidance from CDC was not clear. Remember how they said, “Mask is not necessary?”...Initially they said, “Well, mask is not necessary––only if you are having fever, cough, then you should wear a mask.” That was kind of bad decision they gave us I think about four weeks later, they started saying, “Everyone should wear a mask.” Again, not really sure though. “We recommend everyone.” Now they’re saying, “It’s mandatory everyone should wear a mask.” I think that this coronavirus-19––even CDC did not have a good sense of how it is, and what’s the use of mask and all that. P7 G4

Providers felt they were not able to pass along adequate information to patients, as the information was uneven and, in some cases, conflicting. Likewise, the lack of response from public health authorities left HBCP agencies confused on what care measures were appropriate given the uncertainty around the virus. The lack of evidence or previous understanding of the COVID-19 virus contributed to uncertainty over which preparedness measures were most effective in controlling the adverse health consequences of the pandemic. To cope with this lack of information, HBCP agencies turned to the CDC or their own infection control plans; however, the novelty of the virus left many to question what to do to protect themselves and their patients in the onset of the pandemic.

“The government should do a better job in preparing us to deal with all this. That’s what it is. It’s all coming from them. Whatever they tell us to do—and CMS also. The same thing with CMS because we work with Medicare. It’s all about the guidelines that we follow. [...] Because whatever they think it’s necessary to do—it comes from them. That’s what it is. They have to put out better guidelines, I guess. A better plan, a better preparation. ” P22, G6

“I would say just that we didn't have all the information on the virus. Usually, as nurses, we know what the diagnosis is and what the side effects are and the symptoms are and how to treat it, right? With COVID, it was we didn't have definitive information, right? It was difficult to give information because it was evolving, and we were learning as we was going.” P18 G2

## Discussion

The experiences and perspectives provided by HBCP, here participants employed in Medicare-certified home health agencies, highlight the need for system-wide changes to prevent adverse outcomes and to support older adults to age-in-place during a pandemic. Due to the presence of co-morbidities and social isolation, many patients seen by HBCP providers often require multiple modalities of care, such as increased community support and informal caregiving. Disasters of the scale such as the COVID-19 pandemic are a disruption that can be life-threatening [8, 26, 27]. From the themes discussed above, four areas of potential policy and practice impact were generated – communication, preparedness training, community support, and cultural shifts – that would provide critical support HBCP need to adequately care for their patients during a pandemic.

According to participants, and seen in other studies of home-based care outside of the U.S. [28], messaging was unclear across multiple authorities, from the local to federal level. This led to confusion on what information to relay to their clients and was exacerbated by patient uncertainty over what sources to believe, and in turn, this patient uncertainty aggravated access issues, as providers reported that patients were afraid of contracting the virus due to provider’s visits into the home. Additionally, while agencies were quickly trying to adapt to providing care in a pandemic, guidance from governing bodies such as the Centers for Medicare and Medicaid Services (CMS) and the CDC lagged behind [1, 2, 29].

Participants referenced a lack of consolidated information to pass on to their patients regarding infection prevention and healthy living within the context of a pandemic. This could be mediated by professional organizations or policy advocates in home health and home-based care distilling information from federal resources into palatable sections appropriate for individuals of all literacy levels, which HBCP could then pass on to their patients. Participants also discussed a need for increased flexibility of insurers in times of serious disruption to normal systems of care—the pandemic being a notable example—as well as planning for aging-in-place resources. Insurers such as CMS should increase communication efforts and create exceptions for these contexts. It is crucial for federal agencies such as the CDC and public health officials to critically analyze their information dissemination strategies, as participants overwhelmingly described these as disorganized and confusing. Past studies have shown increased preparedness and organized disaster planning when an effective partnership exists between community health organizations and emergency management offices at the local and federal levels [30].

Our results described that among the home health agencies with pre-pandemic training efforts focused on disaster preparedness and infection control had providers who felt more confident in their ability to maintain care amidst the pandemic. In addition, this readiness enabled them to innovate to develop best practices and adapt to new guidelines. The discrepancy between those who felt prepared and those who described not meeting readiness standards illustrates a need for increased support for preparedness training efforts. Some participants identified the emergency preparedness training mandated by CMS as a facilitator in their ability to maintain the quality of care they provided during the pandemic. However, while CMS requires every organization participating in its programs to comply with the Emergency Preparedness Rule, there exists intentional leeway in how healthcare organizations conduct training and planning [31, 32]. Additionally, these plans have typically focused on preparing HBCP for disasters such as floods, hurricanes, and tornadoes, although training specific to widespread infectious disease processes is a fairly new requirement, occurring just prior to the pandemic [31, 33].

Participants also described a critical shortage of information regarding how the virus spread, level of contagion, and effective prevention and control measures which existed at the initial stages of the pandemic. HBCP organizations may not be incorporated into state-wide emergency preparedness plans, where disaster planning has had a greater focus on hospitals and nursing homes [30, 34]. HBCP need to be prioritized when public health officials consider the dissemination of information due to their vital role in community health care for populations that are most vulnerable to the effects of COVID-19. HBCP share similar goals as emergency managers and disaster response professionals in keeping older adults safe, healthy and in the living environment they desire, making communication and coordination among and between agencies and emergency planners crucial [35].

Due to the pandemic, aspects of community infrastructure patients typically relied on were unavailable, leading to a disruption in aging-in-place. Other studies have demonstrated that social isolation and the abrupt halt of informal support networks such as faith-based or community may contribute to increased adverse health outcomes for older adults [36, 37]. The interruption of these vital sources of support leaves a vacuum which, according to participants, HBCP felt obliged to fill, stepping out of direct care roles in order to meet additional holistic care needs of their patients. Providers tasked to fulfill these vacant roles, previously filled by caregivers, led to feelings of being overwhelmed, overworked, and subsequently burnt out [38]. These findings echo studies of home-based care after Hurricane Harvey, where participants described using informal channels of community support to provide post-hurricane recovery needs [8, 15, 18, 27].

As suggested by participants, innovative ways of using existing community support networks to provide opportunities to socialize and check-in with patients are vital to successful aging-in-place during a pandemic. Collective well-being as a metric for community resilience during disasters is one avenue; if the link between individual and community disaster preparedness is well-established and valued, then disaster-related adverse health effects may be mitigated within a community [39, 40]. This framework can be applied to communal support systems for older adults during pandemic contexts. For instance, participants suggested using existing volunteer organizations to implement monitoring systems to protect against falls and ensure the patient has access to healthy meals and medications. Increased funding for non-profits and non-governmental organizations can support creating and implementing these solutions. By increasing community support through these avenues, the burden of care that falls on any one entity will be redistributed, relieving burnout and allowing for a more holistic care.

The system of home-based care fills a critical gap in community health care and in aging-in-place [41]. Therefore, HBCP must receive the support they need to provide versatile, creative care in ever-changing environments such as the COVID-19 pandemic [42]. This study highlights the feelings of undervalue felt by participants, especially within disaster contexts and is illustrated by the shared consensus that both providers and agencies were viewed as an afterthought by public health authorities, government agencies, and insurance providers, where the focus was on other healthcare organizations such as hospitals and nursing homes [34]. Additionally, participants described the systems-wide challenges they faced, which included financial hardships when trying to meet the CDC’s recommended procedures as well as CMS requirements. For the smaller, privately owned agencies, dipping into reserve supplies placed them under undue financial stress. Many encountered PPE scarcities and staffing shortages due to the perception that HBCP organizations did not serve as vital of a role in COVID-19 response [42, 43]. This lack of recognition, as well as the strain of overtime and working in a resource-poor environment, can lead to caregiver exhaustion and burnout, a concept seen in multiple disaster settings including among staff caring for older adults during Hurricane Katrina [38].

If the shared goal is to help older adults age-in-place during disasters and mitigate health consequences caused by these, home-based care must be valued as an essential facet of community-based care. More accessible physical and mental health resources can give HBCP the bandwidth they need to provide care in the versatile and creative ways necessary in challenging contexts, and even more so during pandemics. Furthermore, consideration for HBCP agencies in PPE allocation and CMS reimbursement for excess cost must be considered, as many are small private agencies and are not able to withstand the financial strain caused by increased resource demand of pandemics. If home-based and community care modalities are prioritized financially and culturally in these ways, some of the most deleterious effects of COVID-19 on older vulnerable populations may be ameliorated.

### Limitations

This study does have limitations that prevent the findings from being widely generalizable. First, the participants came primarily from predominantly from three States in the U.S. This limited the study to a smaller scope of communities with differing sociodemographics. This study does not represent the diverse perspective that is needed to better understand and support structurally marginalized communities. Further rigorously conducted research with a more diverse sample of providers is needed to uncover barriers specific to race, job title, and socioeconomic status [21]. This study draws from the experiences of Medicare-certified home health agencies, and does not represent the views of home-based primary care or long-term care which have different patient-provider relationships. However, our findings are supported by the growing body of literature that gives voice to the important insight of home care providers during the pandemic [42, 43], where we tied our findings to those of the other studies exploring these challenges in the early days of the pandemic.

## Conclusion

Home-based care fulfills an essential need during a disaster, where providers support their clients to maintain continuity of care. This study provides insights on the barriers and facilitators HBCP face while caring for older adults, a population that is particularly susceptible to the dangers disasters inflict on communities. The four themes generated in this study point to larger policy and practice recommendations that could relieve barriers to care, facilitate aging-in-place, and provide vital support for HBCP within disaster contexts. These suggestions can be scaled to be implemented at multiple levels, from local evidence-based practice guidelines to changing federal policy. If resources and political energy are targeted to increase communication, provide needed support for HBCP, bolster community support networks, and recognize the value of home-based care, we will be better prepared for future disasters and pandemics.

## Data Availability

The datasets generated and/or analyzed during this current study are not publicly available due to ongoing use of data set but are available from the corresponding author on reasonable request.
